# Thymic stromal lymphopoietin protects in a model of airway damage and inflammation via regulation of caspase-1 activity and apoptosis inhibition

**DOI:** 10.1038/s41385-020-0271-0

**Published:** 2020-02-26

**Authors:** Nicholas J. Shubin, Morgan Clauson, Kerri Niino, Victoria Kasprzak, Avery Tsuha, Eric Guga, Gauri Bhise, Manasa Acharya, Jessica M. Snyder, Jason S. Debley, Steven F. Ziegler, Adrian M. Piliponsky

**Affiliations:** 1 Center for Immunity and Immunotherapies, Seattle Children’s Research Institute, Seattle, WA 98101 USA; 2 Department of Comparative Medicine, School of Medicine, University of Washington, Seattle, WA 98195 USA; 3 Division of Pulmonary and Sleep Medicine, Seattle Children’s Hospital, Seattle, WA 98105 USA; 4 Immunology Program, Benaroya Research Institute at Virginia Mason, Seattle, WA 98101 USA; 5 Department of Immunology, University of Washington School of Medicine, Seattle, WA 98195 USA; 6 Department of Pediatrics, University of Washington School of Medicine, Seattle, WA 98195 USA; 7 Department of Pathology, University of Washington School of Medicine, Seattle, WA 98195 USA

## Abstract

Thymic stromal lymphopoietin (TSLP), an epithelial cell-derived cytokine, exhibits both pro-inflammatory and pro-homeostatic properties depending on the context and tissues in which it is expressed. It remains unknown whether TSLP has a similar dual role in the airways, where TSLP is known to promote allergic inflammation. Here we show that TSLP receptor (TSLPR)-deficient mice (*Tslpr*^*−/−*^) and mice treated with anti-TSLP antibodies exhibited increased airway inflammation and morbidity rates after bleomycin-induced tissue damage. We found that signaling through TSLPR on non-hematopoietic cells was sufficient for TSLP’s protective function. Consistent with this finding, we showed that TSLP reduces caspase-1 and caspase-3 activity levels in primary human bronchial epithelial cells treated with bleomycin via Bcl-xL up-regulation. These observations were recapitulated in vivo by observing that *Tslpr*^*−/−*^ mice showed reduced Bcl-xL expression that paralleled increased lung caspase-1 and caspase-3 activity levels and IL-1β concentrations in the bronchial-alveolar lavage fluid. Our studies reveal a novel contribution for TSLP in preventing damage-induced airway inflammation.

## Introduction

Thymic stromal lymphopoietin (TSLP) is a member of the IL-2 cytokine family and is a distant paralog of IL-7. TSLP signals via a receptor which includes the IL-7 receptor α-chain (IL-7Rα CD127) and the unique TSLPR chain. TSLPR-deficient mice^1^ have been extensively used to determine the contribution of TSLP-TSLPR signaling to homeostasis and disease. By using this genetic approach, it has been shown that TSLP can play a dual role depending on the context and tissues in which it is expressed: it can be pro-inflammatory in the context of lung and skin allergic disorders^2^ and pro-homeostatic in others, such as colitis^3^. We recently found that patients with sepsis-induced acute respiratory distress syndrome (ARDS) exhibited increased plasma TSLP levels^4^. ARDS is associated with lung epithelial cell loss as well as increased neutrophil recruitment, survival, and mediator release, which are all thought to contribute to poor ARDS outcomes. Despite our knowledge about ARDS pathophysiology, the immunologic processes that can cause and/or prevent these changes are incompletely understood. Based on the evidence that highlights TSLP’s pivotal role in the regulation of inflammation, we were prompted to investigate TSLP’s contribution to the airway inflammatory process associated with tissue damage, a potential mechanism of injury amplification in ARDS^5^.

Using mice with a deficiency in TSLPR^1^ and mice treated with anti-TSLP neutralizing antibodies in a model of bleomycin-induced acute tissue damage and airway inflammation, we discovered that TSLP can reduce inflammation and morbidity rates after bleomycin administration. We further used in vitro and in vivo approaches to show that TSLP’s protective function is mediated by its ability to inhibit apoptosis and caspase-1 activity and, consequently, the production of the caspase-1 substrate and pro-inflammatory cytokine, IL-1β. Collectively, these data are the first to demonstrate that TSLP can play a protective role in airway inflammation by regulating the epithelium’s response to damage.

## Results

### TSLP protects mice from bleomycin-induced airway inflammation

**Fig. 1:**
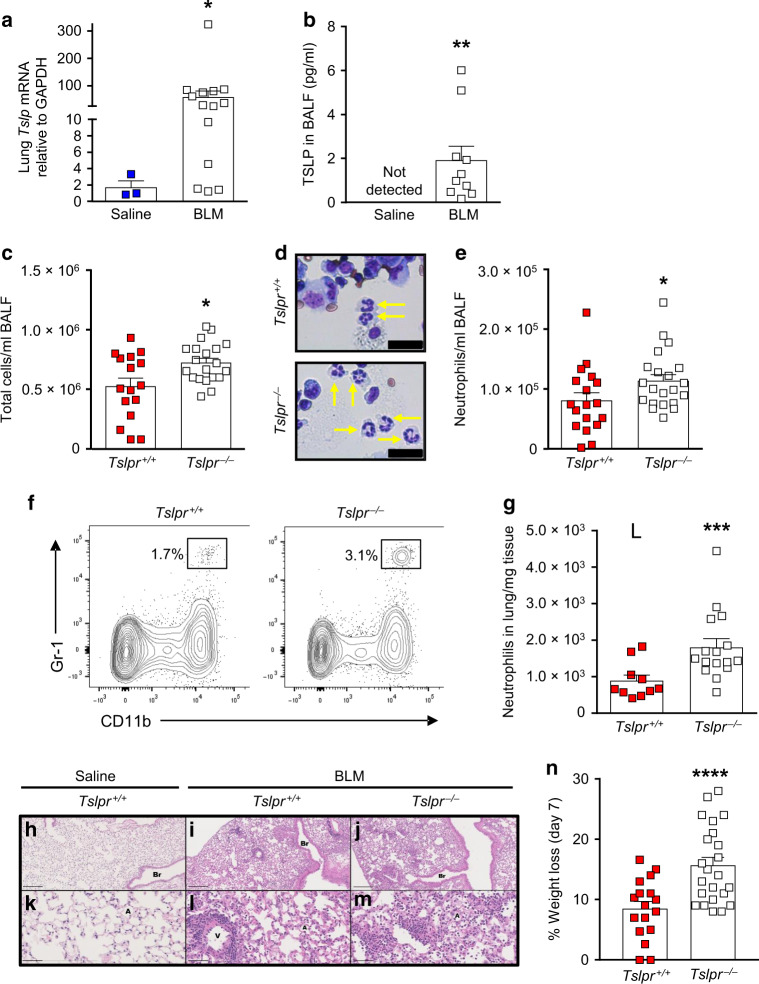
TSLP-TSLPR interactions protect mice from bleomycin-induced airway inflammation. **a**–**n** Mice were administered either sterile saline (pyrogen-free 0.9% NaCl) or bleomycin (100ug) (BLM) on days 1, 3, and 5, and euthanized at day 7. **a**, **b** *Tslp* mRNA expression levels in the lung (**a**) and protein amounts in the BALF (**b**) of C57BL/6 mice. Data were pooled from three independent experiments (*n* = 10–13 mice). *Tslp* mRNA was detectable in 3 out of 10 mice treated with saline. Data are shown as mean + SEM with squares representing values from individual mice. **c**–**g** Total cell numbers in the BALF (**c**), representative BALF cell cytocentrifuge preparations stained with diff-quik (neutrophils are indicated by yellow arrows) (**d**), BALF neutrophil numbers (**e**), representative flow cytometry profile of neutrophils (Gr-1^+^ CD11b^+^) in dissociated lung tissues (**f**) and numbers of neutrophils (Gr-1^+^ CD11b^+^) in the lungs (**g**) of *Tslpr*^*+/+*^ mice (*n* = 10–18) and *Tslpr*^*−/−*^ mice (*n* = 19-20). **h**–**m** Hematoxylin and eosin (HE) histopathology showing that a *Tslpr*^*+/+*^ mouse treated with saline (**h**, **k**) had no changes in its bronchioles or bronchiolar lumens (Br), vessels (V), or alveolar spaces (**a**). *Tslpr*^*+/+*^ mice treated with bleomycin (**i**, **l**) had multifocal mild to moderate perivascular and peribronchiolar inflammation and lower numbers of inflammatory cells within the alveoli. *Tslpr*^*−*/*−*^ mice treated with bleomycin (**j**, **m**) had moderate to regionally severe multifocal inflammation with more severe alveolar neutrophilic inflammation. **h**–**j** bar = 200 µm; Figures **k**–**m**, bar = 50 µm. **n** Weight loss in *Tslpr*^*+/+*^ (*n* = 16) and *Tslpr*^*−/−*^ mice (*n* = 16) are plotted as a percentage of starting weight. Data in **h**–**m** are representative of similar results that were obtained in three independent experiments. Data in **c**, **e**, **g** and **n** were pooled from the five experiments and are shown as mean + SEM with squares representing values from individual mice. *P* value was calculated by Mann–Whitney test. In **a**, **b**, *P < 0.05 and **P < 0.01 versus corresponding values for saline-treated mice. In **c**, **e**, **g** and **n**, *P < 0.05, ***P < 0.005, and ****P < 0.001 versus corresponding values for *Tslpr*^*+/+*^ mice (controls).

It has been shown that injury is one of the main triggers for TSLP release from damaged cells^6^. Therefore, we decided to use a model of bleomycin-induced lung injury, which causes acute tissue damage and airway inflammation^7,8^ to assess the contribution of TSLP to the outcomes of this airway insult. First, we assessed TSLP expression levels in mice at 7 days after oropharyngeal (o.p.) bleomycin administration. A significant increase in TSLP mRNA expression and protein levels in lung tissue and bronchioalveolar fluid (BALF), respectively, was observed in bleomycin-treated mice when compared with saline-treated mice (Fig. 1a, b).

We then assessed the role of TSLP in bleomycin-induced airway inflammation using a genetic approach. For this purpose, we used *Tslpr*^*−/−*^ mice. In comparison with wild type (*Tslpr*^*+/+*^*)* mice, which had a BALF cellularity of 5.3 × 10^5^ ± 6.8 × 10^4^ 7-days post-bleomycin instillation, *Tslpr*^*−/−*^ mice exhibited a marked increase in BALF cellularity (7.2 × 10^5^ ± 3.7 × 10^4^, *n* = 16–20 mice/group, *P* < 0.05) (Fig. 1c). Examination and differential quantification of cyto-centrifuged BALF preparations stained with a modified Wright Giemsa stain revealed increased neutrophilia in *Tslpr*^*−/−*^ mice when compared with *Tslpr*^+/+^ mice (Fig. 1d, yellow arrows; and Fig. 1e). Moreover, the frequency and total number of neutrophils (Gr-1^+^ CD11b^+^) observed by flow cytometric analysis of dissociated lung tissue was significantly greater in *Tslpr*^*−/−*^ mice (Fig. 1f, g). Pathology lung injury scores in *Tslpr*^*+/+*^ mice were significantly lower (range = 8–12; median 9.5, *n* = 6) than in *Tslpr*^*−/−*^ mice (range = 11–17; median 13.5, *n* = 6) after bleomycin administration. *Tslpr*^*−/−*^ mice exhibited more widespread and severe inflammation (Fig. 1j, m) than *Tslpr*^*+/+*^ mice (Fig. 1i, l), with a more pronounced neutrophilic component to the inflammatory infiltrate and greater disruption of the underlying architecture, including expansion of the interstitium by inflammatory cells in the more severely affected mice. In contrast, saline-treated *Tslpr*^*+/+*^ and *Tslpr*^*+/+*^ mice showed only rare perivascular inflammatory cells (Fig. 1h, k). In concordance with the increased neutrophil numbers in the airways, *Tslpr*^*−/−*^ mice exhibited significantly higher BALF and lung expression of the neutrophil chemokine KC when compared with *Tslpr*^+/+^ mice at 7 day after bleomycin administration (fig. S1). Body weight loss, which is associated with increased inflammation and is a good indicator of disease severity, was greater in *Tslpr*^*−/−*^ mice than in *Tslpr*^*+/+*^ mice at 7 day after bleomycin administration (Fig. 1n).

**Fig. 2:**
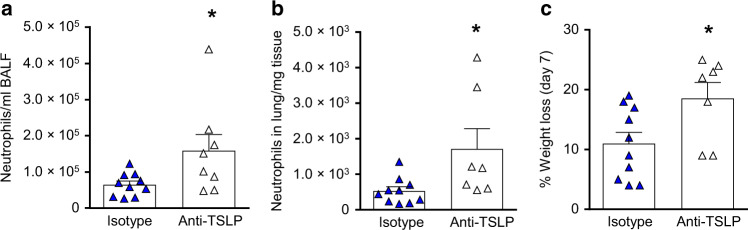
Mice treated with anti-TSLP mAb exhibit increased airway inflammation following bleomycin administration. Mice were administered bleomycin (100 μg) (BLM) on days 1, 3, and 5, and euthanized at day 7. Mice were administered once i.p. with 200 μg anti-TSLP antibody or isotype 24 h prior to bleomycin administration. **a**–**c** BALF neutrophil numbers (**a**), numbers of neutrophils (Gr-1^+^ CD11b^+^) in the lungs (**b**), and weight loss plotted as a percentage of starting weight (**c**) in C57BL/6 mice treated with either isotype (*n* = 10) or anti-TSLP (*n* = 6–8). Data, shown as means + SEM with triangles representing values from individual mice, were pooled from the three independent experiments, each of which gave similar results. *P* value was calculated by Mann–Whitney test. *P < 0.05 versus corresponding values for isotype-treated mice.

Consistent with the phenotype observed in *Tslpr*^*−/−*^ mice, bleomycin-treated C57BL/6 mice that were treated with anti-TSLP neutralizing antibodies exhibited an increase in airway neutrophilia (Fig. 2a, b) and significantly increased body weight loss (Fig. 2c) when compared with bleomycin exposed C57BL/6 mice treated with isotype control.

Numerous studies have shown that the fibrotic phase in the bleomycin-induced acute lung injury model can be observed at 14 day after bleomycin last administration^9^. Accordingly, we scored lung fibrosis on Masson’s trichrome stained lung tissues from mice harvested 14 day following bleomycin administration (Fig. S2A–D). Overall, a trend toward lower fibrosis scores was observed in *Tslpr*^*−/−*^ mice (range = 3–6; mean = 4.5, *n* = 6) when compared with *Tslpr*^*+/+*^ mice (range = 4–6; mean = 5.2, *n* = 5) (Fig. S2E), although this difference was not statistically significant. This observation suggests that TSLP–TSLPR interactions may have a limited impact on the fibrosis phase of the bleomycin-induced acute lung injury model.

To determine whether TSLP–TSLPR interactions can influence acute inflammatory responses in other models, we examined the effects of intranasal (i.n.) LPS administration in *Tslpr*^*−/−*^ mice. In contrast to the bleomycin model, *Tslp*^*+/+*^ and *Tslp*^*−/−*^ mice did not exhibit an increase in TSLP mRNA expression in lung tissues at 6 hours after LPS i.n. administration (Fig. S3A). Moreover, LPS-induced airway inflammation was not influenced by TSLP-TSLPR interactions (Fig. S3B–D). We think that these data are in concordance with previous studies that have shown that primary human airway epithelial cells express low levels of TLR4 and therefore do not release TSLP in response to LPS^10^. In agreement with these studies, we observed that human bronchial epithelial cells (HBECs) obtained from three different donors up-regulate TSLP expression in response to bleomycin or TLR2 and TLR3 ligands used as positive controls, but not LPS (Fig. S4). Based on this evidence, we decided to evaluate the effects of oropharyngeal polyinosine-polycytidylic acid (Poly I:C) administration, a TLR3 agonist, in *Tslpr*^*−/−*^ mice. In comparison with *Tslpr*^*+/+*^ mice, *Tslpr*^*−/−*^ mice exhibited a marked increase in BALF neutrophil numbers and the number of neutrophils in the lungs (Fig. S5A, B). These observations suggest that the down-regulatory effects of TSLP–TSLPR signaling on airway inflammation is restricted to models in which TSLP production is induced, such as the bleomycin and Poly I:C models.

Collectively, these findings indicate that TSLP reduces airway neutrophilic inflammation induced by bleomycin and Poly I:C and protects from bleomycin-induced body weight loss.

### TSLP–TSLPR signaling induces an up-regulation in Bcl-xL expression but does not influence the expression of Th1 and Th2 cytokines in bleomycin-treated mice

Serum albumin concentrations in BALF from *Tslpr*^*−/−*^ mice increased at 7 day after bleomycin oropharyngeal administration but were similar to those found in *Tslpr*^*+/+*^ mice (fig. S6). Therefore, the increased airway inflammatory response in *Tslpr*^*−/−*^ mice cannot be explained by an increase in vascular leak.

**Fig. 3:**
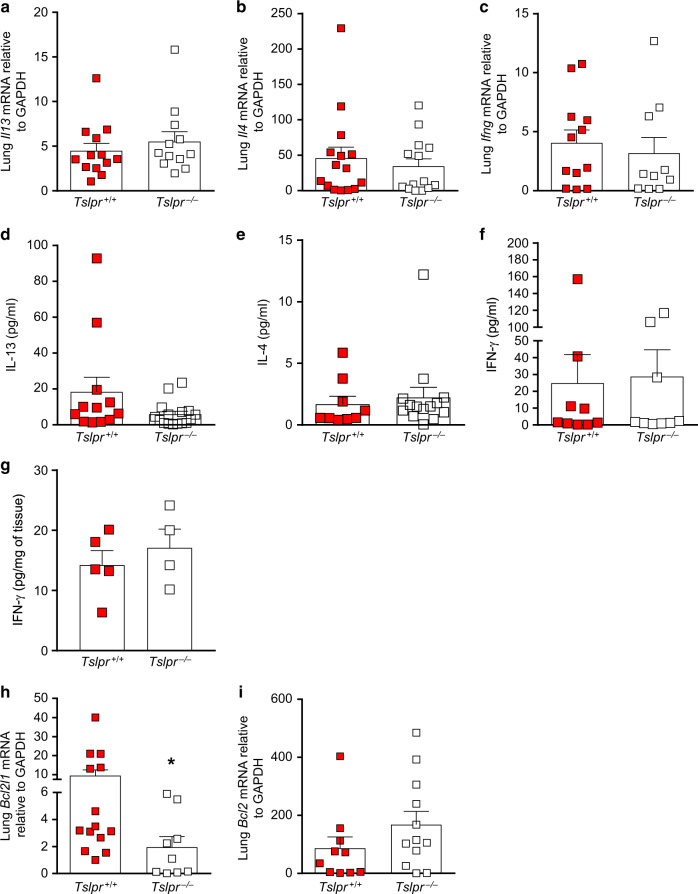
TSLP–TSLPR interactions induce Bcl-xL expression but do not influence the expression of Th1 or Th2-associated mediators in mice during bleomycin-induced airway inflammation. **a**–**f** *Il13, Il4, and Ifng* mRNA expression levels in the lung (**a**–**c**) and protein concentrations in the BALF (**d**–**f**); IFNγ protein concentrations in lung (**g**); (H-I) *Bcl2l1 (Bcl-xL)* (**g**) and *Bcl2* (**h**) mRNA expression levels in the lung in *Tslpr*^*+/+*^ (*n* = 9–15) and *Tslpr*^*−/−*^ mice (*n* = 9–14) 7-days following bleomycin administration (100  μg) (BLM), which was given on days 1, 3 and 5. Lung mRNA expression data in **a**–**c** and **h**–**i** for *Tslpr*^*+/+*^ and *Tslpr*^*−/−*^ bleomycin-treated mice are expressed in relation to their respective saline-treated controls. Data shown as means + SEM with squares representing values from individual mice, were pooled from the three independent experiments, each of which gave similar results. *P* value was calculated by Mann–Whitney test. *P < 0.02 versus corresponding values for *Tslpr*^*+/+*^ mice (controls).

It has been shown that the TSLP-mediated skewing of the T cell response toward a Th2 phenotype and/or suppression of exacerbated Th1 responses can be protective in certain parasitic infections and colitis models^3,11^. Importantly, there is evidence that Th2 cytokines, such as IL-13 and IL-4, whose production can be triggered by TSLP^12,13^, can contribute to reduced bleomycin-induced airway inflammation^14,15^. Based on this evidence, we decided to investigate whether TSLP-TSLPR interactions induce an increase in the Th2 response in bleomycin-treated mice. Our data showed no significant differences in IL-13 and IL-4 mRNA (Fig. 3a, b) and protein concentrations in the BALF (Fig. 3d, e) of *Tslpr*^*+/+*^ and *Tslpr*^*−/−*^ mice treated with bleomycin. Moreover, IFNγ mRNA (Fig. 3c) and protein concentrations in the BALF and lung (Fig. 3f, g) were also similar in *Tslpr*^*+/+*^ and *Tslpr*^*−/−*^ mice. These data suggest that TSLP-TSLPR interactions do not protect from bleomycin-induced inflammation by altering the Th1/Th2 response to injury. Based on these results, we decided to investigate alternative pathways by which TSLP may protect from bleomycin-induced inflammation. Mouse studies showed that TSLP–TSLPR interactions can contribute to CD8 T cell homeostasis and natural helper cell resistance to corticosteroids^16,17^. However, we did not observe a significant reduction in lymphocyte numbers in the BALF from *Tslpr*^*−/−*^ mice when compared with *Tslpr*^*+/+*^ mice (Fig. S7) suggesting that the effects of TSLP on the lymphoid compartment do not contribute to protection from bleomycin-induced airway inflammation.

There is extensive literature supporting a protective role for Bcl-2 and Bcl-xL in models of acute lung injury, including bleomycin-induced damage and inflammation^18–22^. In concordance with this evidence, *Tslpr*^*−/−*^ mice exhibited significantly lower mRNA expression levels for Bcl-xL (Fig. 3h) while a slight, but not significant, increase in Bcl-2 mRNA expression levels (Fig. 3i) was observed when compared with *Tslpr*^*+/+*^ mice treated with bleomycin. These observations indicate that TSLP–TSLPR signaling may mediate its protective effects during bleomycin-induced airway inflammation via Bcl-xL up-regulation.

### TSLP reduces caspase-1 and caspase-3 activity in bleomycin-treated human primary bronchial epithelial cells via Bcl-xL

**Fig. 4:**
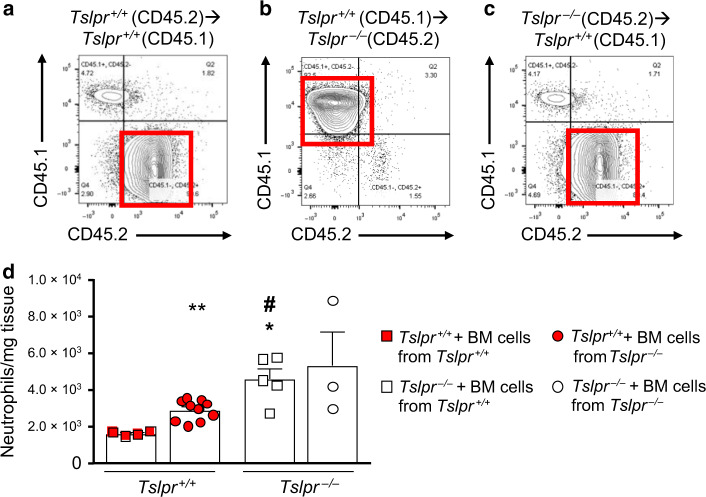
TSLPR activation in structural cells protects from bleomycin-induced airway inflammation. **a**–**c** Peripheral blood analyzed from (**a**) *Tslpr*^*+/+*^ (CD45.1) and (**b**) *Tslpr*^*−/−*^ (CD45.2) recipient mice shows blood chimerism (red box) arising from *Tslpr*^*+/+*^ (CD45.2 or CD45.1) transplanted bone marrow cells. **c** Blood chimerism (red box) in *Tslpr*^*+/+*^ (CD45.1) recipient mice arising from *Tslpr*^*−/−*^ (CD45.2) transplanted bone marrow cells. Data in **a**–**c** are representative of similar results obtained in two independent experiments with 3–5 mice per group. **d** Mice were administered bleomycin (100 μg) (BLM) on days 1, 3, and 5, and euthanized at day 7. Numbers of neutrophils (Gr-1^+^ CD11b^+^ ) in the lungs in *Tslpr*^*+/+*^ (*n* = 4–10) and *Tslpr*^*−/−*^ (*n* = 4–5) mice that received total body irradiation with two exposures of 550 cGy separated by 3–4 h prior to receiving 5 × 10^6^ bone marrow cells from either *Tslpr*^*+/+*^ or *Tslpr*^*−/−*^ mice by i.v. within 4 h of secondary irradiation. Data in **d**, shown as means + SEM with squares representing values from individual mice, were pooled from the two independent experiments, each of which gave similar results. *P* value was calculated by Mann–Whitney test. *P < 0.05 and **P < 0.005 vs. *Tslpr*^*+/+*^ + BM cells from *Tslpr*^*+/+*^; ^#^*P* < 0.05 vs. *Tslpr*^*+/+*^ + BM cells from *Tslpr*^*−/−*^.

Accordingly, we adopted a reductionist approach to dissect mechanisms by which the TSLP-Bcl-xL axis may protect from bleomycin-induced inflammation. For this purpose, we set out to identify potential TSLP target cells during bleomycin-induced inflammation. We have shown that TSLP acts on myeloid cells to down-regulate the inflammatory response in the cecal ligation and puncture (CLP) model of sepsis^4^. Therefore, we used mice in which TSLP–TLSPR signaling is impaired in myeloid cells (*Lys-*Cre^+^; *Tslpr*^*fl/fl*^ mice) to examine whether TSLP reduces inflammation by directly influencing myeloid cell function. As shown in Fig. S8A–C, *Lys-*Cre^+^; *Tslpr*^*fl/fl*^ mice did not show increased bleomycin-induced inflammation and morbidity. Next, we performed studies with mixed bone marrow chimeric mice that included lethally irradiated *Tslpr*^*+/+*^ and *Tslpr*^*−/−*^ mice that were adoptively transferred with bone marrow cells obtained from either *Tslpr*^*+/+*^ or *Tslpr*^*−/−*^ mice. Our analysis of peripheral blood chimerism indicates robust hematopoietic reconstitution of *Tslpr*^*+/+*^ and *Tslpr*^*−/−*^ mice with *Tslpr*^*+/+*^ bone marrow cells (Fig. 4a, b). Moreover, *Tslpr*^*+/+*^ recipient mice could be successfully reconstituted with *Tslpr*^*−/−*^ bone marrow cells (Fig. 4c).

The robust hematopoietic reconstitution in *Tslpr*^*+/+*^ and *Tslpr*^*−/−*^ mice prompted us to evaluate disease severity after bleomycin administration. As shown in Fig. 4d, *Tslpr*^*−/−*^ mice engrafted with either *Tslpr*^*+/+*^ or *Tslpr*^*−/−*^ bone marrow cells exhibited increased neutrophil recruitment in the lung tissue when compared with *Tslpr*^*+/+*^ mice engrafted with either *Tslpr*^*+/+*^ or *Tslpr*^*−/−*^ bone marrow cells suggesting that TSLP mainly targets radio-resistant resident lung cells during bleomycin-induced airway inflammation. It has been shown that human lung epithelial cells constitutively express TSLPR, and stimulation with TSLP can promote epithelial cell growth and wound repair^12^.

These observations led us to hypothesize that TSLP may influence airway epithelial cell function to promote homeostasis and thereby protect against bleomycin-induced inflammation. To test this hypothesis in vitro, we decided to use primary human bronchial epithelial cells (HBECs). HBECs can respond to TSLP stimulus by up-regulating Bcl-xL protein expression levels (Fig. S9) indicating that HBECs are a relevant in vitro system to examine how TSLP-induced Bcl-xL contributes to reduce the effects of bleomycin-induced stress on airway epithelium.

**Fig. 5:**
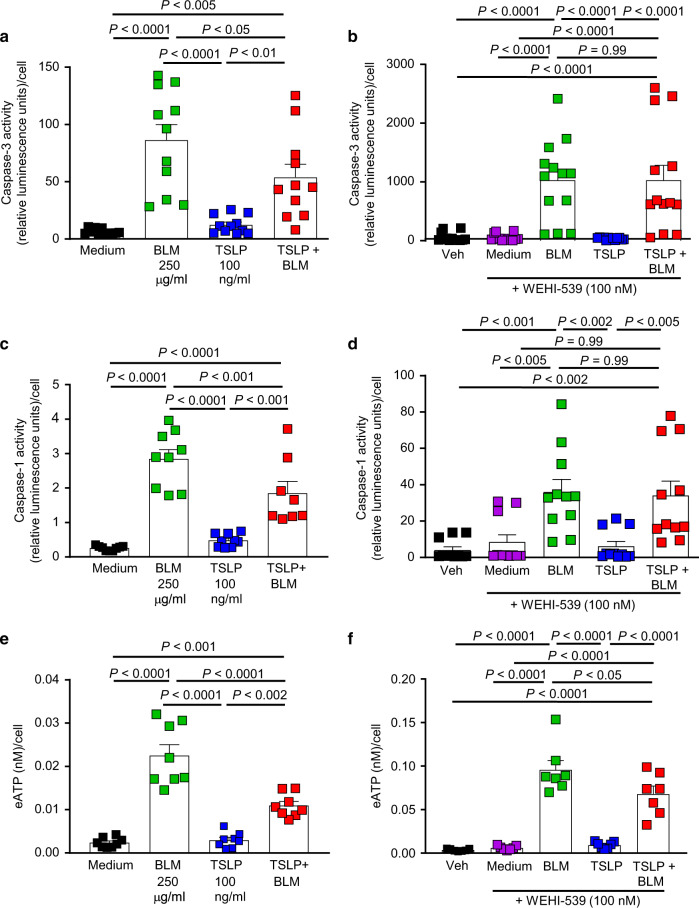
The TSLP-Bcl-xL axis reduces caspase-1 and caspase-3 activity levels, and ATP amounts released by primary human bronchial epithelial cells (HBECs) treated with bleomycin. Caspase-3 (**a**, **b**) and caspase-1 (**c**, **d**) activity levels, and extracellular ATP (eATP) amounts (**e**, **f**) in HBECs treated with or without recombinant human TSLP (100 ng/ml) for 48 h and bleomycin (250 μg/ml) for 24 h (**a**, **c**, **e**); and HBECs treated under the same conditions in the presence of a selective Bcl-xL inhibitor (100 nM) (WEHI-539) or vehicle (Veh) (**b**, **d**, **f**). Data are shown as means + SEM from five independent experiments with squares showing values for individual cell treatments. *P* values were calculated by one-way ANOVA followed by Tukey’s multiple comparisons test.

An association between increased Bcl-xL expression and decreased caspase-3 has been observed in an acute lung injury model that measured lung epithelial cell apoptosis^20^. Importantly, we observed that the addition of TSLP inhibited caspase-3 activity in bleomycin-treated HBECs (Fig. 5a). As shown in Fig. 5b, the inhibitory effect of TSLP on caspase-3 activity levels was partially reversed with the addition of WEHI-539, a potent and selective Bcl-xL inhibitor^23^. It has been shown that Bcl-xL can bind and inhibit the NACHT, LRR, and PYD domains-containing protein (NLRP)1, a key component of the inflammasome complex^24,25^. Notably, NLPR1 promotes caspase-1-mediated IL-1β and IL-18 maturation, which are key pro-inflammatory cytokines in bleomycin-induced lung injury in humans and mice^26^. Based on this evidence we tested whether the protective effects of TSLP in bleomycin-induced inflammation are linked to its ability to inhibit caspase-1 activity via Bcl-xL. Our data indicate that pre-incubation with TSLP for 48 h can reduce caspase-1 activity levels in HBECs treated with bleomycin for 24 h (Fig. 5c), an effect that was significantly inhibited by WEHI-539 addition (Fig. 5d).

A dual role for the NLRP1 and NLRP3 inflammasomes in bleomycin-induced airway inflammation has been reported^27^. The contribution of the NLRP3 inflammasome to caspase-1 activation in bleomycin-induced inflammation is triggered by the release of danger signals, such as ATP, from apoptotic airway epithelial cells^28^. As shown in Fig. 5e, TSLP treatment reduced the extracellular levels of ATP (eATP) generated by HBECs when treated with bleomycin, but this protective effect was significantly attenuated with WEHI-539 addition (Fig. 5f). Overall, these data suggest that TSLP-induced Bcl-xL can inhibit cell apoptosis and prevent caspase-1 activation by reducing the release of danger signals that trigger the activation of the NLRP3 inflammasome.

### The TSLP–TSLPR pathway reduces caspase-1 and caspase-3 activity levels and airway inflammation in bleomycin-treated mice

**Fig. 6:**
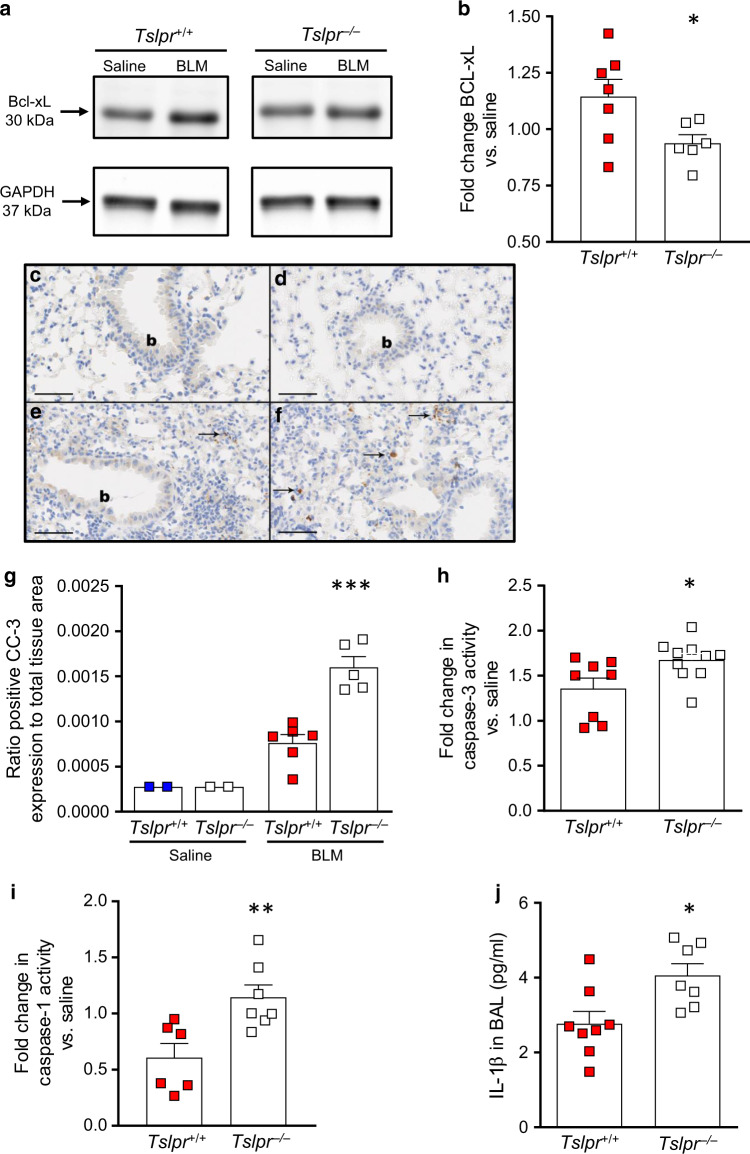
TSLP–TSLPR interactions reduce apoptosis and caspase-1 activity in bleomycin-induced airway inflammation. **a**, **b** Western blot analysis (**a**) and densitometry values for Bcl-xL expression (**b**) in lungs from *Tslpr*^*+/+*^ (*n* = 7) and *Tslpr*^*−/−*^ mice (*n* = 6) at 1 day after either sterile saline or bleomycin (100 μg) (BLM) administration Data in **a** are representative of similar results that were obtained in three independent experiments. Data in **b**, shown as means + SEM with squares representing values from individual mice, were pooled from the three independent experiments, each of which gave similar results. **c**–**g** Cleaved caspase 3 (CC-3) immunohistochemistry (**c**–**f**) and ratio of positive CC-3 expression relative to total tissue area in saline-treated *Tslpr*^*+/+*^ (**c**) and *Tslpr*^*−/−*^ mice (**d**) and at 7 day following bleomycin administration in *Tslpr*^*+/+*^ (**e**) and *Tslpr*^*−/−*^ mice (**f**). Positive anti-cleaved caspase 3 staining is brown (indicated by arrows), with hematoxylin counterstain. Minimal to mild background staining of the bronchiolar epithelium (indicated by “**b**”) is present. **c**–**f** bar = 50 µm. **h** Caspase-3 activity levels in lungs from *Tslpr*^*+/+*^ (*n* = 8) and *Tslpr*^*−/−*^ mice (*n* = 10) at 7 day following bleomycin administration. **i**, **j** Caspase-1 activity levels in lungs (**i**), and IL-1β amounts in the BALF (**j**) of *Tslpr*^*+/+*^ (*n* = 6–8) and *Tslpr*^*−/−*^ mice (*n* = 6–7) at 1 day after bleomycin administration. Data in **g**–**i** were pooled from the three independent experiments and are shown as means + SEM with squares representing values from individual mice. *P* value was calculated by Mann–Whitney test. In **b**, **g**, **h**, **i** and **j**, *P < 0.05, **P < 0.01, and ***P < 0.005 versus corresponding values for *Tslpr*^*+/+*^ mice (controls).

Our in vitro data seem to indicate that TSLP’s protective effects in bleomycin-induced airway inflammation may be explained by its ability to up-regulate Bcl-xL that in turn can prevent cell apoptosis and limit caspase-1 activity and the consequential generation of pro-inflammatory cytokines^26^. To test this hypothesis, we then proceeded to examine whether the suppressive effects of TSLP–TSLPR have an impact on the outcome of bleomycin-induced inflammation. First, we observed that TSLPR-deficient mice were impaired in their ability to up-regulate Bcl-xL protein expression levels in the lung at 1 day after bleomycin administration (Fig. 6a, b). Immunohistochemistry studies for the cleaved or active form of caspase-3 (CC-3) showed a significantly increased ratio of positive CC-3 to total tissue area in lung tissues from *Tslpr*^*−/−*^ mice treated with bleomycin (*n* = 6) compared with *Tslpr*^*+/+*^ mice (*n* = 6) (Fig. 6c–g). Moreover, caspase 3 activity levels in the lung tissue of *Tslpr*^*−/−*^ mice were significantly increased when compared with *Tslpr*^*+/+*^ mice treated with bleomycin (Fig. 6h).

Reduced Bcl-xL expression levels were also associated with increased caspase-1 activity levels in the lungs of *Tslpr*^*−*/*−*^ mice when compared with *Tslpr*^+/+^ mice at 1 day after bleomycin administration (Fig. 6i). In bleomycin-challenged mice, it has been shown that increased caspase-1 activity results in increased mature IL-1β levels^26^. Importantly, IL-1β is required for the increased neutrophil recruitment into the bronchoalveolar space of bleomycin-challenged mice^29^. In agreement with these reports, we found that IL-1β levels were increased in *Tslpr*^*−/−*^ mice that also exhibited increased caspase-1 activity levels at 1d after bleomycin administration (Fig. 6j).

Together, these data suggest that TSLP-TSLPR signaling induces Bcl-xL up-regulation, which reduces cell apoptosis and bleomycin-induced airway inflammation caused by increased caspase-1 and caspase-3 activity in this setting.

## Discussion

TSLP has been studied extensively in the context of lung and skin allergic disorders where it promotes inflammatory Th_2_ responses^30^; however, it is becoming clear that TSLP also influences other disorders and multiple organ systems, including accelerated wound healing in inflammatory bowel disease (IBD), promotion of cancer, and exacerbation of autoimmunity^30^. Importantly, mouse models of these disorders such as IBD, have shown that TSLP can play a beneficial role by limiting inflammation in response to damage and/or by promoting wound healing^3,11^. ARDS is characterized by potent inflammatory responses within the lungs that result in widespread damage to the alveolar-capillary barrier, flooding of the airspaces with protein-rich edema fluid, and severe gas-exchange abnormalities^31^. Recently, we observed increased plasma TSLP levels in sepsis-induced ARDS patients^4^. Based on our own research that TSLP can reduce inflammation and morbidity in the CLP model of sepsis^4^, we hypothesized that TSLP may also play a protective role in a mouse model of damage-induced inflammation that resembles the early inflammatory stages of ARDS. For this purpose, we treated mice with a deficiency in the TSLP-specific TSLPR subunit required for TSLP signaling^1^ with bleomycin, a medication that can induce lung tissue damage and inflammation. We found that TSLPR-deficient mice exhibited increased lung injury, inflammation in the airways and morbidity after bleomycin administration (Fig. 1). A similar phenotype was observed with the administration of anti-TSLP antibodies to C57BL/6 mice (Fig. 2) confirming that TSLP-TSLPR ligation confers protection in a model of damage-induced airway inflammation.

As a Th2 cytokine, TSLP might well be expected to alter matrix deposition and fibrosis. However, we did not observe evidence for a significant TSLP-mediated pro-fibrotic effect at 14 day after bleomycin administration (Fig. S2). This observation suggests that TSLP-TSLPR interactions can reduce inflammation with a reduced impact on the fibrosis phase of the bleomycin-induced acute lung injury model. A similar phenotype has been recently reported for mice deficient in the inducible T cell costimulatory molecule (ICOS) involved in type 2 inflammatory responses^32^. Like TSLP, ICOS protects against bleomycin-induced acute lung injury without having a pro-fibrotic effect^32^. Based on this evidence, we think that Th2-associated mediators/molecules can down-regulate inflammation without promoting lung fibrosis after bleomycin administration.

In contrast to our observations, Herro et al. reported a significant reduction in the fibrosis phase of bleomycin-induced acute lung injury in *Tslpr*^*−/−*^ mice when compared with *Tslpr*^*+/+*^ mice^33^. We can only speculate at this point that well-known factors that can influence bleomycin potency such as the route of administration (i.t. vs. o.p.)^34^ contributed to the development of acute lung injury models of different severities and TSLP concentrations in the airways that may or may not be enough to induce fibrosis in tissues.

The next question in our study was to understand the mechanisms by which TSLP exerts its protective role in the airways upon bleomycin-induced damage. It is well-known that TSLP can trigger the production of Th2 cytokines, such as IL-13 and IL-4. Despite the critical role of these cytokines in the promotion of allergic inflammation, IL-13 and IL-4 can reduce bleomycin-induced airway inflammation^14,15^. However, we did not observe that TSLP–TSLPR signaling influences IL-4 and IL-13 expression in the airways (Fig. 3a, b), instead, we found that TSLP–TSLPR signaling induces an up-regulation in the expression of the anti-apoptotic molecule Bcl-xL (Fig. 3d). By using a genetic approach, we showed that Bcl-xL up-regulation is associated with reduced apoptosis and caspase-1 activity levels. By reducing caspase-1 activity and the consequential generation of pro-inflammatory cytokines such as IL-1β (Fig. 6), TSLP–TSLPR signaling can limit the magnitude airway inflammatory response triggered by bleomycin. This finding is consistent with Bcl-xL’s known ability to bind and inhibit NLRP1, a component of the inflammasome that leads to pro-IL-1β cleavage into mature IL-1β via caspase-1 activation. Interestingly, TSLP production can be induced by IL-1β^35,36^, suggesting the existence of a TSLP-mediated negative feedback loop that can limit the magnitude of the inflammatory response to injury.

TSLP’s ability to protect against bleomycin-induced inflammation by limiting the amounts of mature IL-1β generated by caspase-1 is particularly relevant to our understanding of ARDS pathogenesis considering the extensive amount of literature supporting a detrimental role for this pro-inflammatory cytokine in this disorder^37–41^. It has been shown in vitro that IL-4 can suppress NLRP3-dependent caspase-1 activation and the subsequent IL-1β secretion by macrophages in a transcription-independent manner^42^. However, there is no evidence that IL-4 or other Th2-associated mediators use a similar mechanism of protection to what we report here for TSLP in in vivo models of damage-induced inflammation. In fact, there is very limited information on the cellular and molecular mechanisms by which Th2-associated mediators protect in these models. For example, it has been shown that IL-4 and IL-5 are anti-inflammatory in models of bleomycin-induced airway inflammation by regulating T cell and myeloid cell expansion, respectively^15,32^ but the molecular mechanisms are unknown.

By using mixed bone marrow chimeric mice, we found that neutrophil recruitment in the lung tissue in response to bleomycin largely depends on TSLP–TSLPR signaling in radio-resistant resident lung cells (Fig. 4e). It is important to point out, though, that we did observe a significant increase in lung neutrophilia in *Tslpr*^*+/+*^ mice that received *Tslpr*^*−/−*^ bone marrow cells when compared with *Tslpr*^*+/+*^ mice that received *Tslpr*^*+/+*^ bone marrow cells. This suggests a partial contribution of hematopoietic cells expressing TSLPR to protection from bleomycin-induced airway inflammation. Studies are under way to attempt to identify these hematopoietic cells targeted by TSLP in our model.

The fact that human lung epithelial cells constitutively express TSLPR and stimulation with TSLP promotes epithelial cell growth and wound repair^12^ led us to hypothesize that airway epithelial cells may be one of the main targets for TSLP upon bleomycin-induced damage. We provided evidence that supports our hypothesis by showing that the TSLP-Bcl-xL axis reduced caspase-1 activity in HBECs upon bleomycin treatment (Fig. 5c). Interestingly, our in vitro studies also showed that TSLP-induced Bcl-xL can reduce the release of danger signals from apoptotic cells that can mediate the activation of the NLRP3 inflammasome and the consequent increase in caspase-1 activity levels (Fig. 5c–f). However, our in vivo data showed that TSLP–TSLPR signaling does not significantly influence the BALF levels of danger signals, such as eATP and uric acid (Fig. S10). These data suggest that the in vivo environment may provide additional pro-homeostatic signals that may reduce airway epithelial cell susceptibility to bleomycin-induced damage and apoptosis, and hence excessive caspase-1 activation via the NLRP3 inflammasome. For example, endothelial cells have been shown to significantly contribute to homeostasis in acute lung injury by secreting mediators with known anti-apoptotic effects on the endothelium itself and epithelium, such as vascular endothelial growth factor (VEGF)^43^. Despite this evidence, we cannot rule out a more significant contribution of TSLP to the inhibition of caspase-1 activation via the NLRP3 inflammasome in other models of airway inflammation where the NLRP3 may play a more dominant role, such as with rhinovirus^44^ and *Pseudomonas aeruginosa*^45^ infection, as well as exposure to cigarette and biomass fuel smoke^46^.

Overall, this study provides additional novel evidence to the growing body of work reporting that mediators and cells typically associated with the development of airway inflammation in the context of chronic Th2 disorders, such as allergy and asthma, can be protective in sepsis and damage-induced airway inflammation^4,32,47–49^. Like other Th2-associated mediators with protective effects in bleomycin-induced inflammation, it remains unclear how TSLP can have a dual role in Th2-mediated disorders, such as asthma and allergy, and bleomycin-induced inflammation. Overall, the data available in the literature seem to suggest that the pro-homeostatic effects of TSLP might be tissue specific (mainly in the gastrointestinal tract) and context specific (parasitic infections and colitis), and manifest within a narrow window of concentrations^50^. Another plausible explanation for this paradox is that the short and long forms of TSLP (hereafter called sfTSLP and lfTSLP, respectively), which can exhibit anti-inflammatory and pro-inflammatory properties in the airways^51^, respectively, are differentially up-regulated during allergic inflammation and inflammation induced by cell damage. However, this possibility may only explain the dual effect of TSLP in the human system, as the sfTSLP isoform has not been yet described in mice^50^.

Our in vitro studies showed that TSLP can protect primary human airway epithelial cells from bleomycin-induced damage; however, the relevance of our data to human disease remains unknown. We recently reported that TSLP levels were increased in sepsis-induced ARDS^4^ patients, but the small number of patients tested did not allow us to statistically establish whether TSLP levels in these patients associated with clinical outcomes of the disease. We think that additional studies in this regard are imperative to establish whether patients receiving anti-TSLP therapy for the treatment of asthmatic or allergic disorders may be at higher risk of developing ARDS induced by sepsis, injury, or other causes.

## Materials and methods

### Mice

C57BL/6 mice were purchased from Jackson Laboratories. *Tslpr*^*−/−*^ mice on the C57BL/6 background were previously described^1^. Mice with transgenic expression of *Lys*-Cre on the C57BL/6 background were purchased from Jackson laboratories and crossed with mice containing loxP-flanked *Tslpr* alleles^52^. Mice were bred and maintained at the Seattle Children’s Research Institute Animal Facility. Unless specified otherwise, all experiments were performed using male or female mice that were 12 weeks old at the beginning of the experiment. All animal care and experimentation was conducted in accordance with the current National Institutes of Health guidelines and with the approval of the Seattle Children’s Research Institute Institutional Animal Care and Use Committee.

### Bone marrow chimeras

*Tslpr*^*+/+*^ and *Tslpr*^*−/−*^ mice received total body irradiation with two exposures of 550 cGy separated by 3–4 h prior to receiving 5 × 10^6^ bone marrow cells from either *Tslpr*^*+/+*^ or *Tslpr*^*−/−*^ mice by i.v. within 4 h of the second irradiation. Mice were placed on Baytril antibiotic water (0.5 mg/ml). Baytril treatment began up to 3 days prior to irradiation and continued for 3 weeks post-irradiation. Experiments were performed with the mouse chimeras 7–8 weeks after adoptive transfer of bone marrow cells.

Peripheral blood chimerism was examined to assess the robustness of hematopoietic reconstitution in *Tslpr*^*+/+*^ and *Tslpr*^*−/−*^ mice. For this purpose, *Tslpr*^*+/+*^ (CD45.1 genotype) and *Tslpr*^*−/−*^ (CD45.2 genotype) recipient mice were lethally irradiated as described above and received bone marrow cells from *Tslpr*^*+/+*^ mice of CD45.2 or CD45.1 genotype, respectively. A separate group of *Tslpr*^*+/+*^ mice (CD45.2 genotype) was reconstituted with *Tslpr*^*−/−*^ bone marrow cells (CD45.1 genotype). Peripheral blood was obtained at 7–8 weeks after irradiation and white blood cells were then stained using antibodies to CD45.1 and CD45.2 and analyzed by flow cytometry. Levels of blood chimerism were calculated as the proportion of CD45 labeled white blood cells that expressed the donor CD45.1 or CD45.2 genotypes.

### Bleomycin-induced airway inflammation

Briefly, mice were anesthetized via isoflurane inhalation and then oropharyngeal (o.p) administered 100ug of bleomycin sulfate (catalog number BML-AP302, Enzo Life Sciences, Farmingdale, NY) in 25ul sterile saline (pyrogen-free 0.9% NaCl) on days 1, 3, and 5. For morbidity assessment, the mice were observed at least three times daily for the first day, and then twice a day for up to fourteen days. Additionally, the mice were weighed daily. Mice were euthanized if weight loss was above 20% from initial body weight or they were clearly moribund. Data from mice that exhibited weight loss above 20% shown in Figs. 1n and S2C were obtained at the planned euthanasia time (7 days after first bleomycin administration). After 1 or 7 days, the mice were euthanized and the lungs were flushed through an incision in the trachea with 1 mL PBS for BALF collection. The lungs were then flushed an addition four times with PBS for BALF cell collection. Then, portions of the right lungs were collected for RNA, protein, and flow cytometry evaluations.

For histological assessment, the trachea, thymus, heart, and left lungs were collected, a ligation was made between the heart and left lungs, and the lungs were inflated with 10% saline-buffered formaldehyde. Fixed tissues were paraffin embedded, cut into ~4 µm sections, and stained with hematoxylin and eosin (H&E). Pulmonary changes were scored semi-quantitatively by a board-certified veterinary pathologist, not blinded to genotype. Briefly, numerical scores were assigned based on the maximum degree of severity of inflammation in the alveolar space; bronchiolar lumen; interstitium; and perivascular space, with 0 = normal; 1 = few inflammatory cells; 2 = larger foci of inflammatory cells, with preservation of underlying architecture; 3 = larger foci of inflammatory cells with mild changes of underlying vessel, alveolar wall or bronchiolar epithelium; 4 = larger foci of inflammatory cells with disruption or loss of underlying architecture including necrosis and/or hemorrhage. Each compartment was scored individually, and a total inflammatory score was also assigned for global lung inflammation based on similar criteria. A percent lung inflammation involvement score was assigned with 0 = normal; 1 = ≤5%; 2 = 6–10%; 3 = 11–20%; and 4 = >20% pulmonary parenchymal involvement. Bronchiolar epithelium was graded for hyperplasia and inflammation. A cumulative score was calculated, with a maximum possible score of 28.

In a separate experiment, mice were euthanized at 14 days for Masson’s Trichrome staining of lung tissues and fibrosis evaluation. Lung fibrosis scoring was performed using slight modifications of a previously described scoring system^53^. The pathologist scoring the slides was blinded to the genotype and treatment. Briefly, lungs were assigned a severity score from 0 (normal) to 4 (marked) and an extent score from 1 (occasional alveolar duct and bronchioles) to 4, with the total score representing the severity score times the extent score.

Images of representative lesions were acquired using NIS-Elements BIR 3.2 64-bit or directly from digitally scanned images and plated in Adobe Photoshop Elements. Image brightness and contrast was adjusted using AutoSmart Fix and/or Auto levels (White Balance) manipulations applied to the entire image.

For treatment with the anti-TSLP antibody, mice were i.p. injected once with 200 μg anti-TSLP antibody (clone M702, Amgen) or control IgG 24 h prior to bleomycin administration.

### Statistical analyses

All statistics were performed using Prism software (GraphPad Software, Inc., La Jolla, CA). Mann–Whitney *U*-test, Wilcoxon Signed Rank Test and one-way ANOVA followed by Tukey’s multiple comparisons test were performed as noted in the respective figure legends. *P* < 0.05 was considered statistically significant. Unless otherwise specified, all data are presented as mean + SEM.

## Supplementary information

Supplemetary Methods

Supplemental Figure 1

Supplemental Figure 2

Supplemental Figure 3

Supplemental Figure 4

Supplemental Figure 5

Supplemental Figure 6

Supplemental Figure 7

Supplemental Figure 8

Supplemental Figure 9

Supplemental Figure 10
