# Supplementary material for: Thymic stromal lymphopoietin protects in a model of airway damage and inflammation via regulation of caspase-1 activity and apoptosis inhibition
Source: Mucosal Immunol. 2020 Feb 26;13(4):584–94. doi: 10.1038/s41385-020-0271-0 (PMC7312418; doi:10.1038/s41385-020-0271-0)
Supplement: Supplementary file 1 — Supplemetary Methods [file 41385_2020_271_MOESM1_ESM.docx]

**Supplementary Methods**

**LPS-induced airway inflammation**

Briefly, mice were anesthetized via isoflurane inhalation, and 10 μg LPS from *Escherichia coli* O55:B5 (catalog number L4005, Sigma-Aldrich) diluted in 25 μl of sterile saline was instilled i.n. After 6 hours, the mice were euthanized, and the lungs were flushed through an incision in the trachea with 1mL PBS for BALF collection. The lungs were then flushed an addition 4 times with PBS for BALF cell collection. Then, portions of the right lungs were collected for RNA and flow cytometry evaluations.

**Poly: IC-induced airway inflammation**

Mice were anesthetized via isofluorane inhalation and then oropharyngeal administered 50 μg of poly: IC, high molecular weight (catalog number tlrl-pic, Invivogen, San Diego, CA). Mice were given poly: IC on day 0 and day 1 and were euthanized at day 3 for BALF and tissue collection.

**Flow cytometry**

Lungs were first lavaged to collect BALF, which removed all circulating cells from the lungs. The lower right lung lobe was collected, minced, and incubated in a digestion media containing DNase I (10 kunitz units/mL; catalog number D4263, Sigma Aldrich, St. Louis, MO) and Collagenase A (1mg/mL; catalog number 10103578001, Sigma Aldrich), 5% fetal bovine serum (catalog number 10438026, Gibco/Fisher Scientific, Waltham, MA), and penicillin-streptomycin (catalog number 15140163, Gibco/Fisher Scientific) in RPMI 1640 at 37^o^C for 45 min. Following washes with RPMI 1640 and PBS, single cell suspensions were stained with a combination of the following antibodies: Alexa Fluor 700-conjugated mAb to Ly-6G/Ly-6C (clone RB6-8C5, catalog number 108421, BioLegend, San Diego, CA); eFluor 450-conjugated mAb to CD11b (clone M1/70, catalog number 48-0112-82, Thermo Fisher Scientific); allophycocyanin (APC)-conjugated mAb to F4/80 (clone BM8, catalog number 123115, BioLegend); and fluorescein isothiocyanate (FITC)-conjugated mAb to CD45 (clone 30-F11, catalog number 103107, BioLegend).

White blood cells from mouse chimeras were stained with a combination of the following antibodies: PE-Cy7-conjugated mAb to CD45.1 (clone A20, catalog number 110729, BioLegend) and APC-Cy7-conjugated mAb to CD45.2 (clone 104, catalog number 109823, BioLegend). 4', 6-diamidino-2-phenylindole (DAPI) (catalog number D9542, Sigma-Aldrich) was used to detect dead cells. Only cells that were negative for DAPI were used for the cell population analysis.Cells were acquired on a BD LSR II with FACSDiva software and analyzed with FlowJo software (version 10, Tree Star).

**BALF cell evaluations**

Following BALF collection, samples were spun down and resuspended in PBS+5% FBS. Approximately 2 x 10^5^ cells were centrifuged onto glass slides with a cytospin and then slides were stained with the Diff-Quik staining kit (catalog number B4132-1A, Siemens, Newark, DE). Then, at least 2 random-200X images were taken of the slides (Leica DM600R microscope, Leica DFC300X camera, and Leica LAS X software). At least 100 cells were counted, and macrophage, neutrophil and lymphocyte percentages were determined based on cell morphology**.**

**Cytokine** **and chemokine** **measurements**

Concentrations for mouse serum albumin, TSLP, KC, and IL-1β were measured in the BALF with ELISAs. The serum albumin ELISA was from Abcam (Cambridge, MA) (catalog number ab207620); TSLP ELISA was from R&D Systems (Minneapolis, MN) (catalog number MTLP00); the KC ELISA was from Peprotech (Rocky Hill, NJ) (catalog number 900-K127); and the IL-1β ELISA was from Invitrogen/ThermoFisher (catalog number BMS6002). The detection limits for these assays were 25 pg/ml for serum albumin, 6.3 pg/ml for TSLP, 4.0 pg/ml for KC and 1.2 pg/ml for IL-1β. Concentrations for IL-13, IL-4 and IFNγ were measured in the BALF and lung tissues with a bead-based immunoassay from BioLegend (catalog number 740029) and flow cytometry. The detection limits for this assay were 2.1 pg/ml for IL-13, 0.9 pg/ml for IL-4 and 1.1 pg/ml for IFN

**Immunohistochemistry**

Immunohistochemistry was performed using a rabbit polyclonal antibody to mouse cleaved caspase-3 (CC-3) diluted 1:250 (clone D3E9, catalog number 9579, Cell Signaling) on formalin-fixed paraffin embedded tissues. Slides were deparaffinized on the Leica Bond Automated Immunostainer (Leica Microsystems, Buffalo Grove, IL). Antigen retrieval with EDTA was performed at 100°C for 20 min. All subsequent steps were performed at room temperature. Blocking consisted of Leica peroxide block for 5 min followed by an additional blocking step with 10% normal goat serum (catalog number 005-000-121, Jackson ImmunoResearch Laboratories) in tris-buffered saline for 20 min. The primary antibody against CC-3, was diluted in Leica Primary Antibody Diluent and was applied for 30 minutes. A peroxidase-conjugated secondary antibody, goat polyclonal anti-rabbit IgG was then applied for 8 minutes. Antibody complexes were visualized using Leica Bond Mixed Refine (DAB, 3,3'-diaminobenzidine) detection 2X for 10 min. Tissues were counterstained with hematoxylin.

Slides were scanned in brightfield with a 20X objective using a NanoZoomer Digital Pathology System (Hamamatsu City, Japan). The digital images were then imported into Visiopharm software (Hoersholm, Denmark) for analysis. Using the Visiopharm Image Analysis module, regions of interests (ROI) were manually drawn around lung tissue in each image. By converting the initial digital image into grayscale values using two initial features, RGB-R and HDAB-HDAB, the Visiopharm software was trained to label positive staining, CC-3, and background tissue counterstain, hematoxylin, using a project specific configuration based on a threshold of pixel values. The images were processed in batch mode using this configuration to generate the desired per area outputs and analyzed at 100%.

**qPCR**

RNA (1 μg) was isolated from cells with a RNeasy mini kit (catalog number 74104, Qiagen, Redwood City, CA) and converted to first-strand cDNA with the iScript™ cDNA Synthesis Kit (catalog number 1708890, BioRad, Hercules, CA). cDNA was analyzed for quantitative expression levels of *Tslp, Il1b, Kc, Bcl2, Bcl2l1 (Bcl-xL), ll13, Il4,* or *Ifng* in mouse tissue and *TSLP* in HBECswith the Maxima™ SYBR Green/ROX qPCR Master Mix (catalog number K-0222, ThermoFisher) on a 7500-Fast-Real-Time PCR System or a Step One Plus Real-Time PCR System Thermal Cycling Block (Applied Biosystems). The primers used were as follows: *Tslp forward* 5’- CCCTTCACTCCCCGACAAAA -3’, *Tslp reverse* 5’- CGTCATTTCTCTCAGTTTCAGGG -3’; *Il1b *forward 5’- GCCCATCCTCTGTGACTCAT -3’, *Il1b * reverse 5’- AGGCCACAGGTATTTTGTCG -3’; *Kc forward* 5’- TGGCTGGGATTCACCTCAAG -3’, *Kc reverse* 5’- CCGTTACTTGGGGACACCTT -3’; *Bcl2 forward* 5’- GACTGAGTACCTGAACCGGC -3’, *Bcl2 reverse* 5’- AGTTCCACAAAGGCATCCCAG -3’; *Bcl2l1* *forward* 5’- GGCCTTTTTCTCCTTTGGCG -3’, *Bcl2l1 reverse* 5’- AGCACCTCACTCAATGGCTC -3’; *Il13 forward*

5’- CCTGGCTCTTGCTTGCCTT -3’, *Il13 reverse* 5’- GGTCTTGTGTGATGTTGCTCA -3’; *IL4 forward* 5’- GGGACGCCATGCACGGAGATG -3’, *Il4 reverse* 5’- TGCGAAGCACCTTGGAAGCCC -3’; and *Ifng forward* 5’- ACTGGCAAAAGGATGGTGAC -3’, *Ifng reverse* 5’- TGAGCTCATTGAATGCTTGG -3’; *TSLP* forward 5’- TAGAGCCGCAGGCACCCTCT-3’, *TSLP* reverse 5’- TGGGTTGCCCTTCCCACTGGT-3’. Results were analyzed using the dCt method normalized to *Gapdh/GAPDH*. Lung mRNA expression data for *Tslpr^+/+^* and *Tslpr^-/-^* bleomycin-treated mice are expressed in relation to their respective saline-treated controls. Messenger RNA expression data for HBECs are expressed in relation to vehicle-treated cells.

**Western blot analysis**

Lung samples were incubated with T-PER protein extraction reagent (catalog number 78510, ThermoFisher) supplemented with a cocktail of protease inhibitors and EDTA (Halt protease inhibitor cocktail, catalog number 78430, ThermoFisher), homogenized, and then spun down to collect soluble protein lysate. Samples were then denatured by boiling for 10 min at 70 °Cwith 1X sample buffer (NuPage 4X LDS sample buffer, catalog number NP0007, Invitrogen/ThermoFisher) and 1X reducing agent (NuPAGE 10X sample reducing agent, catalog number NP0009, Invitrogen/ThermoFisher) and separated with SDS/PAGE (4-20% Bis-Tris-gels, catalog number M42012, Genscript, Piscataway, NJ) with MOPS running buffer (catalog number M00138, Genscript), electroblotted onto Invitrolon polyvinylidene difluoride membranes (catalog number NP0315, Invitrogen/ThermoFisher), and then probed with antibodies against Bcl-xL (clone 54H6, catalog number 2764, Cell Signaling, Danvers, MA) or GAPDH as a loading control (clone GA1R, catalog number MA5-15738, ThermoFisher). Densitometry was evaluated with the NIH ImageJ software.

**Primary human bronchial epithelial cell (HBEC) culture and treatments**

Primary HBEC samples were obtained from healthy children with 4-mm Harrell unsheathed bronchoscope cytology brushes (ConMed Corp, Utica, NY) and maintained in culture as described^1^. Healthy children ages 6-18 years who were undergoing an elective surgical procedure that required general anesthesia were recruited. Subjects lacked a history of asthma, any other chronic lung disease, prematurity (birth < 36 weeks gestation), allergic rhinitis, atopic dermatitis, or history of a positive skin prick test or positive radioallergosorbent testing (RAST) for a common aeroallergen.

Written informed consent was obtained from a parent or legal guardian for all subjects below the age of 18 years, or from the subject if age 18 years. In addition, written assent was obtained for children ≥ age 10 years. This work was approved by the Seattle Children’s Hospital Institutional Review Board.

HBECs were treated with or without recombinant human TSLP (100 ng/ml, catalog number 300-62, Peprotech) for 48 h and bleomycin(250 μg/ml) for 24 h.In a separate set of experiments,HBECs were treated in the same conditions in the presence of a selectiveBcl-xL inhibitor, WEHI-539 (catalog number A3935, Apexbio, Houston, TX), (100 nM) or vehicle (DMSO)**.**

**Caspase 1 activity assays**

HNEC samples were evaluated for caspase-1 activity levels with a luminescent assay (Caspase-1 Glo 1 Inflammasome Assay, catalog number G9951, Promega, Madison, WI). Mouse lung samples were evaluated for caspase 1 activity levels with a fluorescent assay (Caspase 1 Fluoremetric Assay Kit, catalog number K110, Biovision, Milpitas CA).

**Caspase 3 activity assays**

Caspase 3 activity assays were used to evaluate for apoptosis in cells and tissues. HNEC samples were evaluated for caspase-3 activity levels with a luminescent assay (Caspase-3/7 Glo kit, catalog number G8090, Promega). Mouse lung samples were evaluated for caspase-3 activity levels with a fluorescent assay (CaspGLOW Fluorescein Active Caspase-3 Staining Kit, catalog number K183, Biovision).

**Extracellular ATP levels**

Extracellular ATP levels were measured with a luminescence assay (ATPlite 1step Luminescence Assay System, catalog number 6016736, Perkin Elmer, Waltham, MA).

1. Lopez-Guisa JM, Powers C, File D, Cochrane E, Jimenez N, Debley JS. Airway epithelial cells from asthmatic children differentially express proremodeling factors. *J Allergy Clin Immunol* 2012; **129**(4)**:** 990-997 e996.
