# Supplementary material for: Thymic stromal lymphopoietin protects in a model of airway damage and inflammation via regulation of caspase-1 activity and apoptosis inhibition
Source: Mucosal Immunol. 2020 Feb 26;13(4):584–94. doi: 10.1038/s41385-020-0271-0 (PMC7312418; doi:10.1038/s41385-020-0271-0)
Supplement: Supplementary file 2 — Supplemental Figure 1 [file 41385_2020_271_MOESM2_ESM.pdf]

Supplemental Figure 1

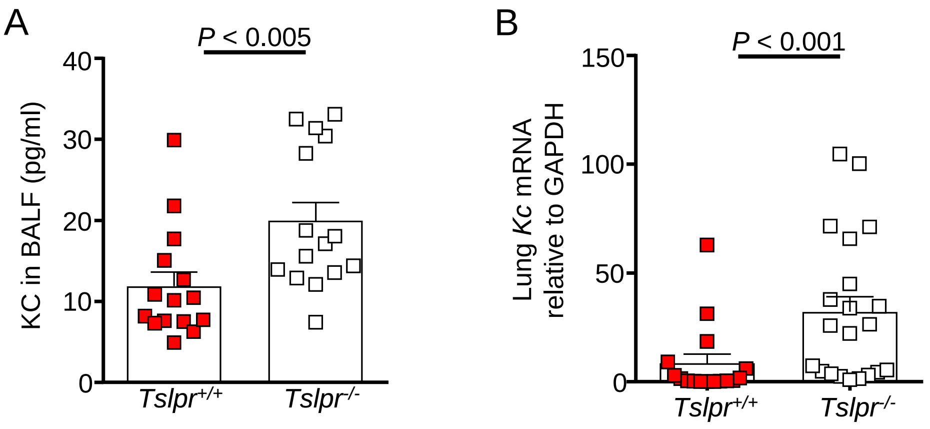

Supplemental Figure 1. KC levels are increased in *Tslpr*$^{-/-}$ mice following bleomycin administration. (A-B) BALF KC amounts (A) and *Kc* mRNA expression levels in the lungs of *Tslpr*$^{+/+}$ *(n* = 14-15) and *Tslpr*$^{-/-}$ mice *(n* = 15-21) administered with bleomycin (100ug) (BLM) on days 1, 3, and 5, and euthanized at day 7. Data, shown as means + SEM with squares representing values from individual mice, were pooled from 4 independent experiments, each of which gave similar results.
