# Supplementary material for: Thymic stromal lymphopoietin protects in a model of airway damage and inflammation via regulation of caspase-1 activity and apoptosis inhibition
Source: Mucosal Immunol. 2020 Feb 26;13(4):584–94. doi: 10.1038/s41385-020-0271-0 (PMC7312418; doi:10.1038/s41385-020-0271-0)
Supplement: Supplementary file 3 — Supplemental Figure 2 [file 41385_2020_271_MOESM3_ESM.pdf]

Supplemental Figure 2

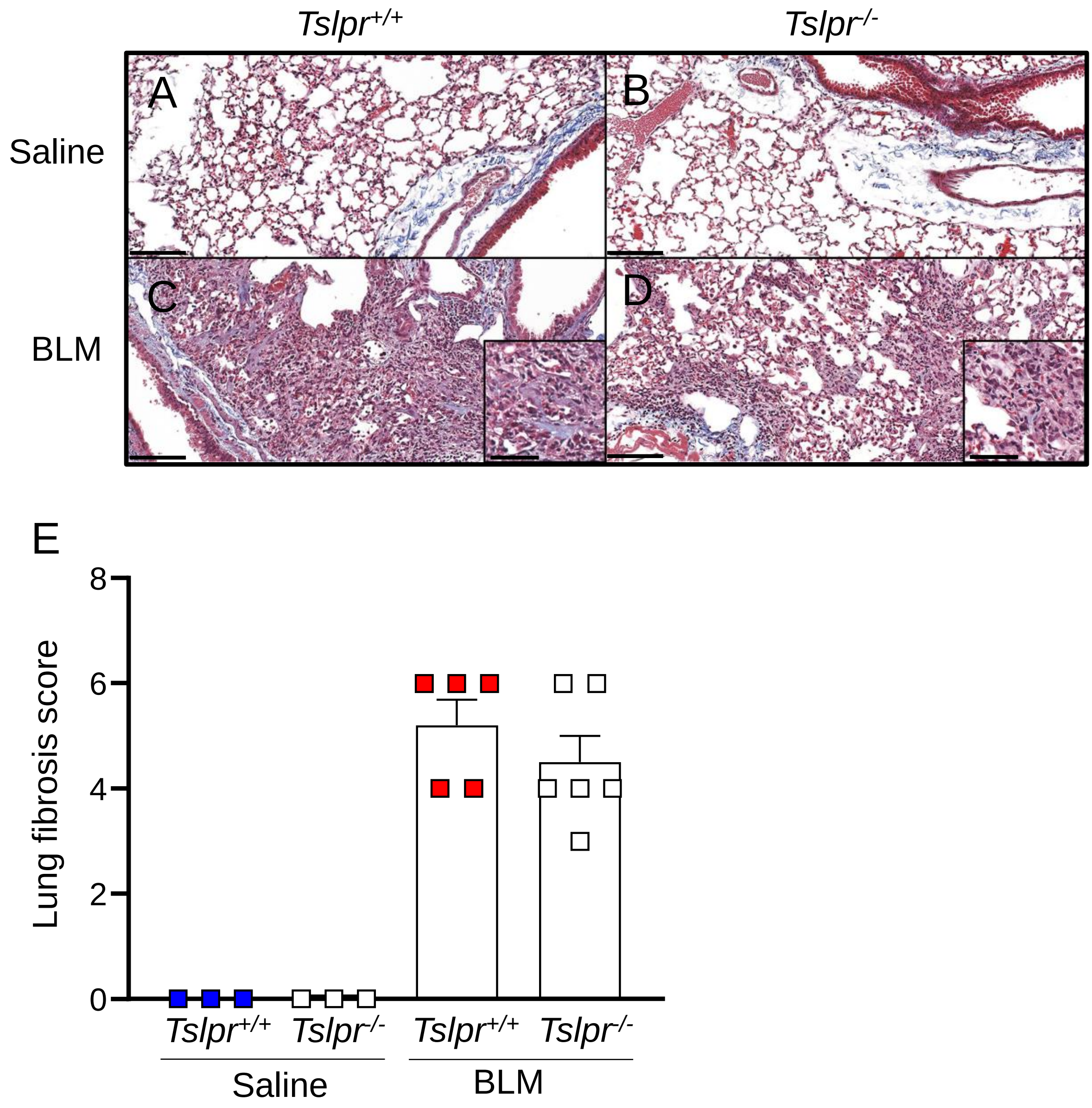

Supplemental Figure 2. TSLP-TSLPR interactions do not influence lung collagen deposition in mice after bleomycin administration. (A-D) Histochemical staining for fibrosis (Masson trichrome stain, collagen shown in blue) in saline treated *Tslpr*$^{+/+}$ (A) and *Tslpr*$^{-/-}$ mice (B) and 14 days following bleomycin administration in *Tslpr*$^{+/+}$ (C, inset) and *Tslpr*$^{-/-}$ mice (D, inset). Collagen is normally present around the vessels and, to a lesser extent, bronchioles, as seen in the saline treated mice. Figures A-D, bar=100 μm and insets, bar=50 μm. (E) Lung fibrosis score as described in Materials and Methods. Data in A-D are representative of similar results that were obtained in 2 independent experiments. Data in E were pooled from 2 experiments and are shown as mean + SEM with squares representing values from individual mice.
