# Supplementary material for: Thymic stromal lymphopoietin protects in a model of airway damage and inflammation via regulation of caspase-1 activity and apoptosis inhibition
Source: Mucosal Immunol. 2020 Feb 26;13(4):584–94. doi: 10.1038/s41385-020-0271-0 (PMC7312418; doi:10.1038/s41385-020-0271-0)
Supplement: Supplementary file 4 — Supplemental Figure 3 [file 41385_2020_271_MOESM4_ESM.pdf]

Supplemental Figure 3

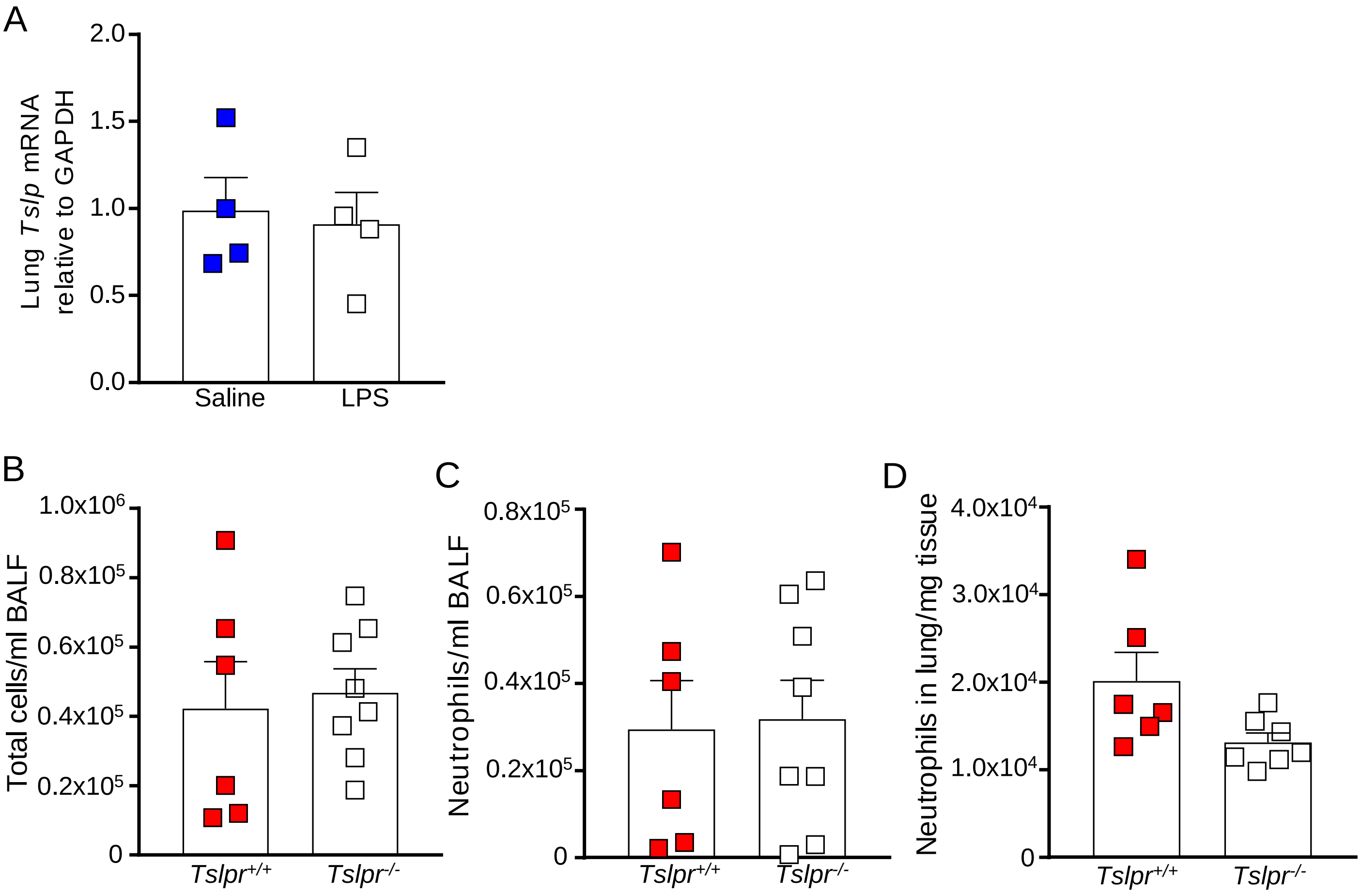

Supplemental Figure 3. TSLP-TSLPR interactions do not influence LPS-induced airway inflammation. (A-D) Mice were administered either sterile saline (pyrogen-free 0.9% NaCl) or LPS (10ug) and euthanized 6 h later. (A) *Tslp* mRNA expression levels in the lung of C57BL/6 mice. Data were pooled from 2 independent experiments (*n* = 4 mice). Data are shown as mean + SEM with squares representing values from individual mice. (B-D) Total cell numbers in the BALF (B), BALF neutrophil numbers (C) and numbers of neutrophils (Gr-1^{+} CD11b^{+}) in the lungs (D) of *Tslpr*$^{+/+}$ mice (*n* = 6) and *Tslpr*$^{-/-}$ mice (*n* = 7). Data in B-D are representative of similar results that were obtained in 2 independent experiments and are shown as mean + SEM with squares representing values from individual mice.
