# Supplementary material for: Thymic stromal lymphopoietin protects in a model of airway damage and inflammation via regulation of caspase-1 activity and apoptosis inhibition
Source: Mucosal Immunol. 2020 Feb 26;13(4):584–94. doi: 10.1038/s41385-020-0271-0 (PMC7312418; doi:10.1038/s41385-020-0271-0)
Supplement: Supplementary file 5 — Supplemental Figure 4 [file 41385_2020_271_MOESM5_ESM.pdf]

Supplemental Figure 4

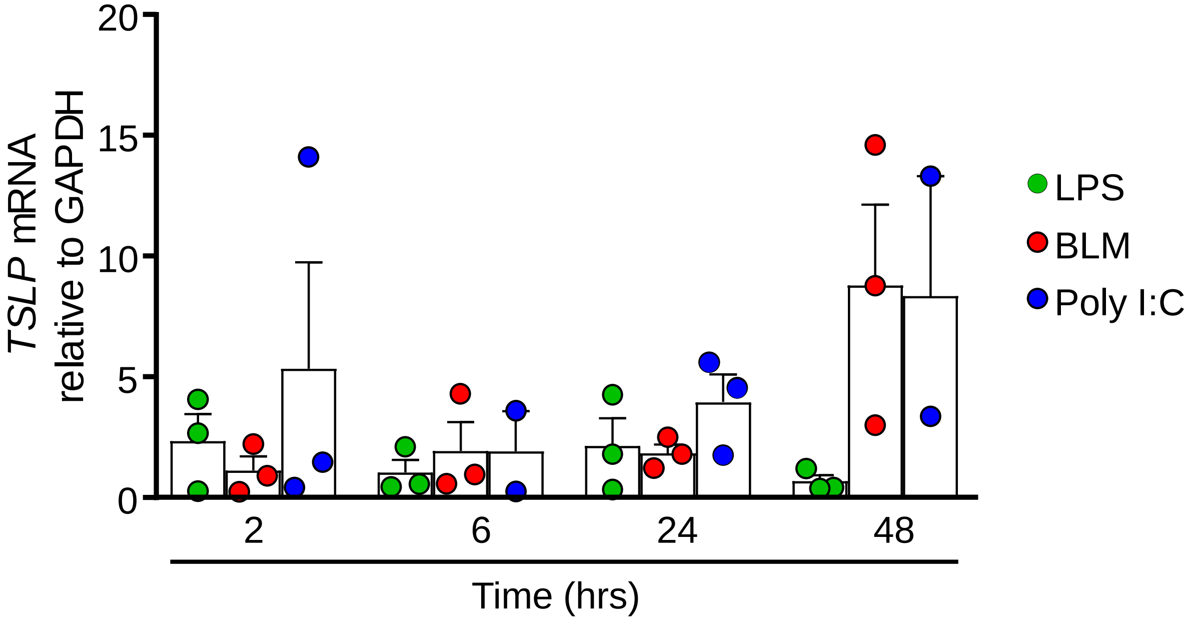

Supplemental Figure 4. Bleomycin but not LPS induces TSLP expression in primary human bronchial epithelial cells (HBECs). HBEC mRNA expression for *TSLP* upon treatment with either LPS (1 ug/mL), bleomycin (BLM) (250 ug/mL) or poly:IC (20 ug/mL) for the indicated time points. Data, shown as means + SEM with circles representing values from cells obtained from individual donors, were pooled from the 3 independent experiments.
