# Supplementary material for: Thymic stromal lymphopoietin protects in a model of airway damage and inflammation via regulation of caspase-1 activity and apoptosis inhibition
Source: Mucosal Immunol. 2020 Feb 26;13(4):584–94. doi: 10.1038/s41385-020-0271-0 (PMC7312418; doi:10.1038/s41385-020-0271-0)
Supplement: Supplementary file 6 — Supplemental Figure 5 [file 41385_2020_271_MOESM6_ESM.pdf]

Supplemental Figure 5

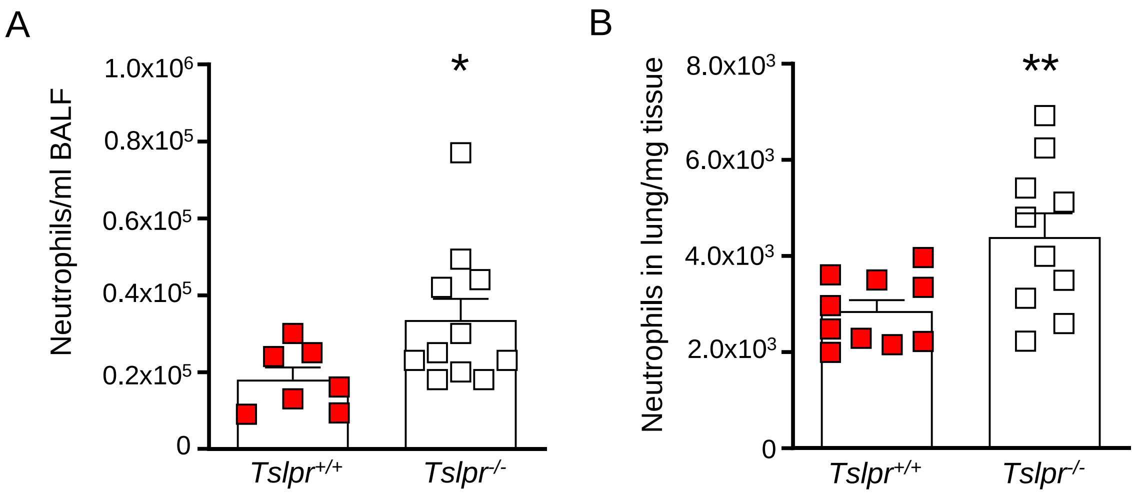

Supplemental Figure 5. TSLP-TSLPR interactions attenuate neutrophil infiltration into lungs in response to Poly I:C administration. L (A-B) Mice were administered either sterile saline (pyrogen-free 0.9% NaCl) or Poly I:C (50ug) on day 0 and day 1 and euthanized at day 3. (A-B) BALF neutrophil numbers (A) and numbers of neutrophils (Gr-1^+ $CD11b^+$) in the lungs (B) of *Tslpr*$^{+/+}$ mice (n = 7-10) and *Tslpr*$^{-/-}$ mice (n = 10-11). Data in A-B are representative of similar results that were obtained in 3 independent experiments and are shown as mean + SEM with squares representing values from individual mice. P value was calculated by Mann Whitney test. In Figs. A and B, * $P < 0.05$ and ** $P < 0.02$ versus corresponding values for *Tslpr*$^{+/+}$ mice (controls).
