# Supplementary material for: Thymic stromal lymphopoietin protects in a model of airway damage and inflammation via regulation of caspase-1 activity and apoptosis inhibition
Source: Mucosal Immunol. 2020 Feb 26;13(4):584–94. doi: 10.1038/s41385-020-0271-0 (PMC7312418; doi:10.1038/s41385-020-0271-0)
Supplement: Supplementary file 7 — Supplemental Figure 6 [file 41385_2020_271_MOESM7_ESM.pdf]

Supplemental Figure 6

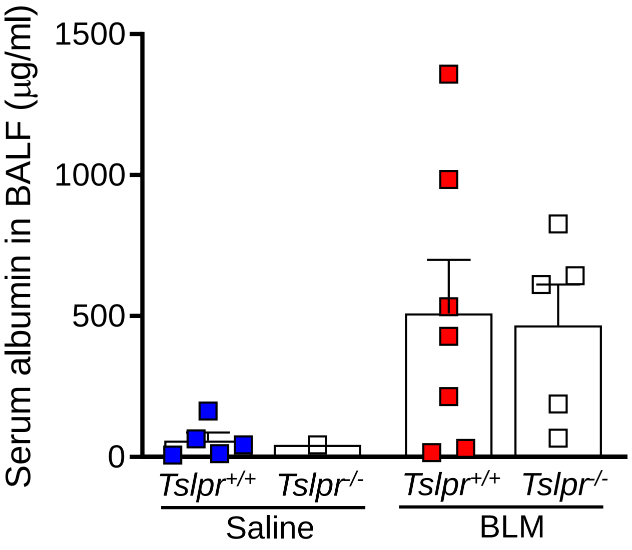

Supplemental Figure 6. Serum albumin concentrations in BALF after bleomycin administration. Mice were administered either sterile saline (pyrogen-free 0.9% NaCl) or bleomycin (100ug) (BLM) on days 1, 3, and 5, and euthanized at day 7. Data were pooled from the 3 experiments and are shown as mean + SEM with squares representing values from individual mice.
