# Supplementary material for: Thymic stromal lymphopoietin protects in a model of airway damage and inflammation via regulation of caspase-1 activity and apoptosis inhibition
Source: Mucosal Immunol. 2020 Feb 26;13(4):584–94. doi: 10.1038/s41385-020-0271-0 (PMC7312418; doi:10.1038/s41385-020-0271-0)
Supplement: Supplementary file 8 — Supplemental Figure 7 [file 41385_2020_271_MOESM8_ESM.pdf]

Supplemental Figure 7

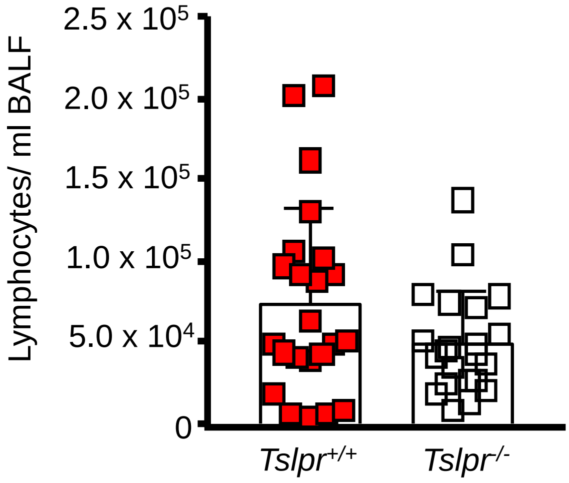

Supplemental Figure 7. TSLP-TSLPR interactions do not influence lymphocyte numbers in mice during bleomycin-induced airway inflammation. Lymphocyte numbers in the BALF of *Tslpr*$^{+/+}$ mice (*n* = 23) and *Tslpr*$^{-/-}$ mice (*n* = 22). Data in were pooled from the 5 experiments and are shown as mean + SEM with squares representing values from individual mice.
