# Supplementary material for: Thymic stromal lymphopoietin protects in a model of airway damage and inflammation via regulation of caspase-1 activity and apoptosis inhibition
Source: Mucosal Immunol. 2020 Feb 26;13(4):584–94. doi: 10.1038/s41385-020-0271-0 (PMC7312418; doi:10.1038/s41385-020-0271-0)
Supplement: Supplementary file 9 — Supplemental Figure 8 [file 41385_2020_271_MOESM9_ESM.pdf]

Supplemental Figure 8

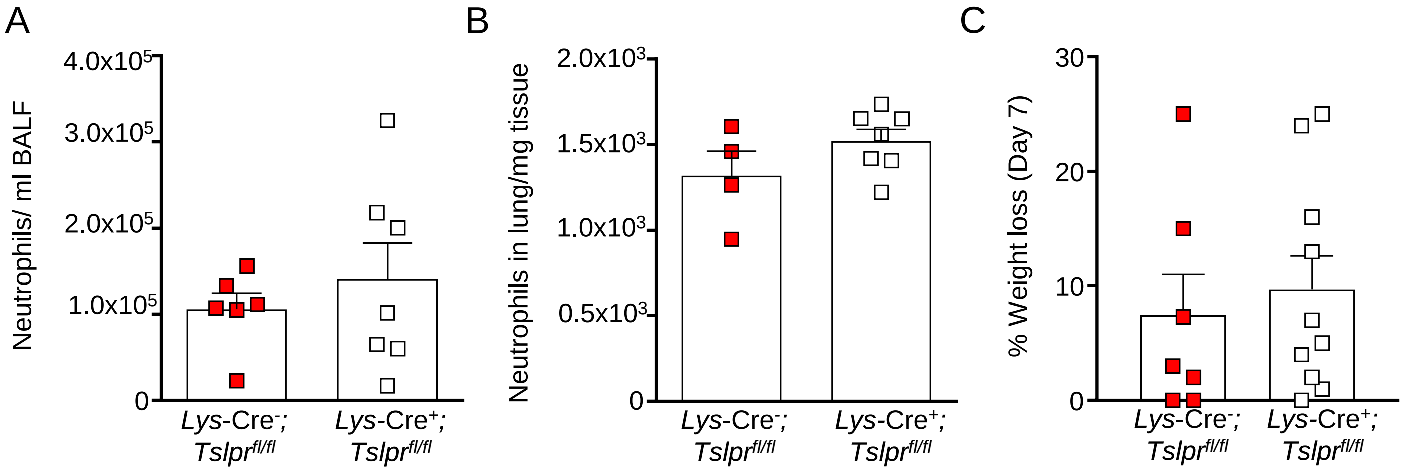

Supplemental Figure 8. TSLPR activation in myeloid cells does not protect from bleomycin-induced airway inflammation. Mice were administered bleomycin (100ug) (BLM) on days 1, 3, and 5, and euthanized at day 7. BALF neutrophil numbers (A), numbers of neutrophils (Gr-1^{+} CD11b^{+}) in the lungs (B) and weight loss, plotted as a percentage of starting weight (C), in *Lys*-Cre^{-}; *Tslpr*$^{fl/fl}$ (*n* = 4-7) and *Lys*-Cre^{+}; *Tslpr*$^{fl/fl}$ mice (*n* = 7-10). Data, shown as means + SEM with squares representing values from individual mice, were pooled from the 2 independent experiments, each of which gave similar results.
