# Supplementary material for: Thymic stromal lymphopoietin protects in a model of airway damage and inflammation via regulation of caspase-1 activity and apoptosis inhibition
Source: Mucosal Immunol. 2020 Feb 26;13(4):584–94. doi: 10.1038/s41385-020-0271-0 (PMC7312418; doi:10.1038/s41385-020-0271-0)
Supplement: Supplementary file 10 — Supplemental Figure 9 [file 41385_2020_271_MOESM10_ESM.pdf]

Supplemental Figure 9

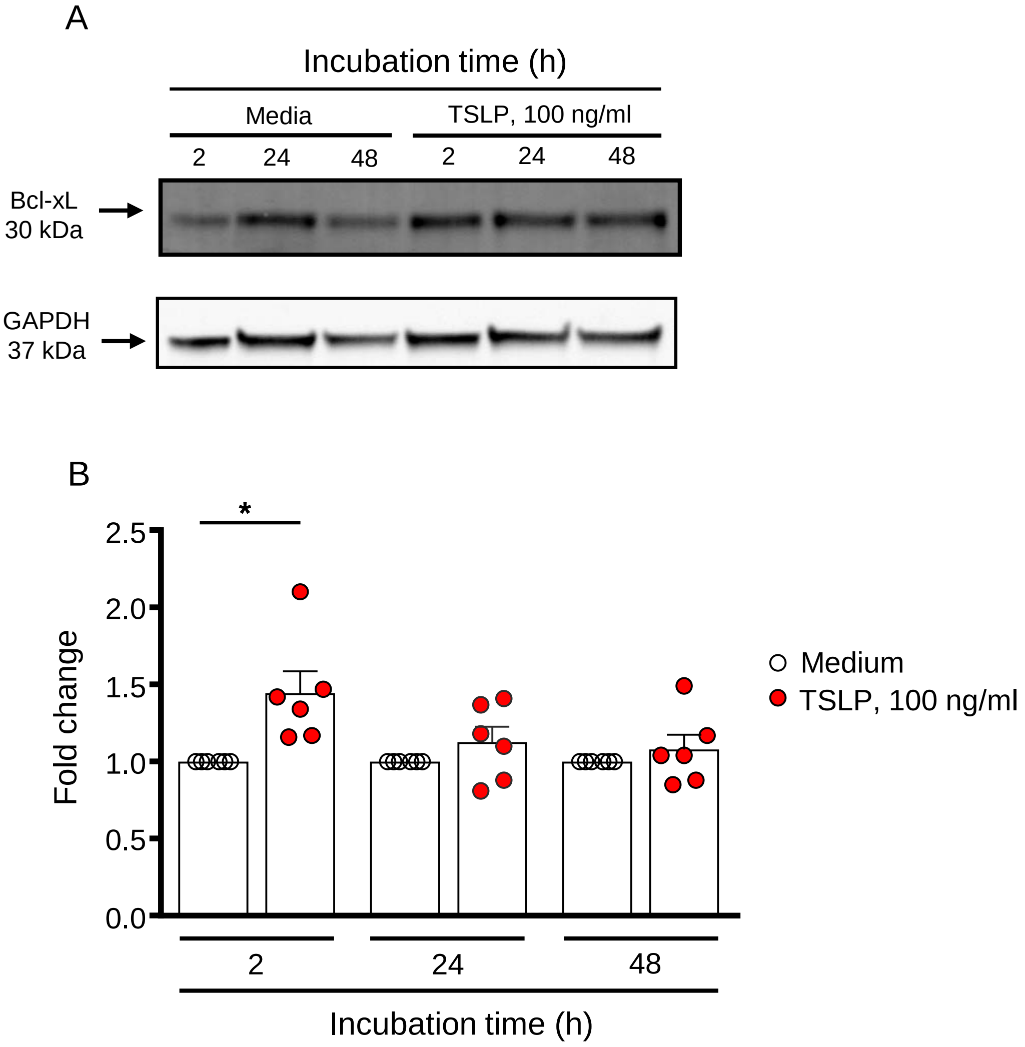

Supplemental Figure 9. TSLP induces Bcl-xL expression in primary human bronchial epithelial cells (HBECs). (A-B) HBECs were treated with medium or human recombinant TSLP (100 ng/ml) for the indicated time points. Western blot analysis (A) and densitometry values (B) for Bcl-xL protein expression. Data are representative of the similar results that were obtained in 6 independent experiments. *P* value was calculated by Wilcoxon Signed Rank Test. * $P < 0.05$ versus corresponding values for medium-treated cells.
