# Supplementary material for: Thymic stromal lymphopoietin protects in a model of airway damage and inflammation via regulation of caspase-1 activity and apoptosis inhibition
Source: Mucosal Immunol. 2020 Feb 26;13(4):584–94. doi: 10.1038/s41385-020-0271-0 (PMC7312418; doi:10.1038/s41385-020-0271-0)
Supplement: Supplementary file 11 — Supplemental Figure 10 [file 41385_2020_271_MOESM11_ESM.pdf]

Supplemental Figure 10

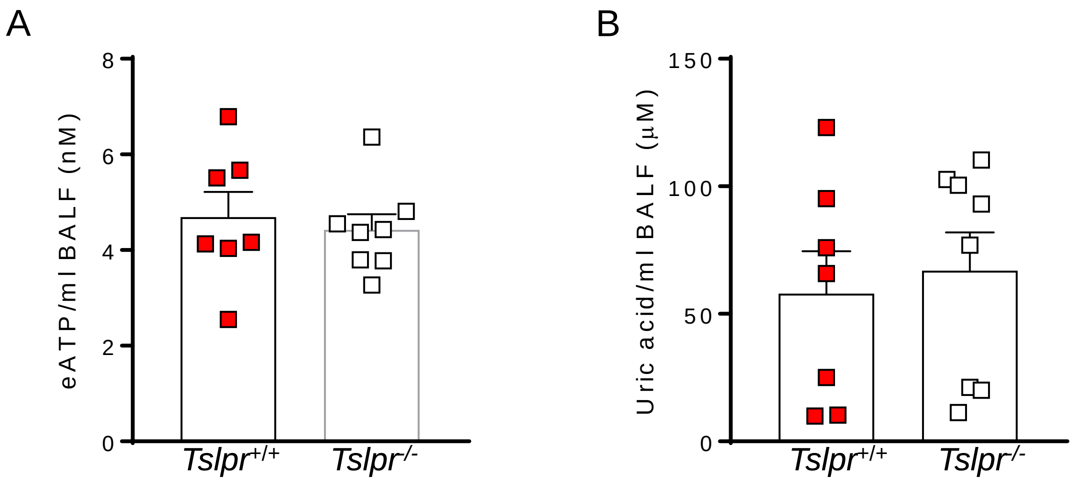

Supplemental Figure 10. TSLP-TSLPR signaling does not influence extracellular ATP (eATP) and uric acid levels generated during bleomycin-induced airway inflammation. eATP (A) and uric acid (B) levels in the BALF of *Tslpr*$^{+/+}$ mice (*n* = 7) and *Tslpr*$^{-/-}$ mice (*n* = 8) obtained at 4h and 1d after one bleomycin (100ug) (BLM) o.p. administration for eATP and uric acid determinations, respectively. Data, shown as means + SEM with squares representing values from individual mice, were pooled from the 2 independent experiments, each of which gave similar results.
